# Guillain-Barre Syndrome Secondary to the Use of Dabrafenib and Trametinib for the Treatment of Advanced Thyroid Carcinoma

**DOI:** 10.7759/cureus.35069

**Published:** 2023-02-16

**Authors:** Jaskaran Batra, Anvitha Ankireddypalli, Ashok K Kanugula, Swathi Gorle, Jasleen Kaur

**Affiliations:** 1 Department of Internal Medicine, University of Pittsburgh Medical Center (UMPC) McKeesport, McKeesport, USA; 2 Department of Endocrinology, University of Minnesota School of Medicine, Minneapolis, USA; 3 Department of Internal Medicine, WellStar Health System-Splading Regional Hospital, Griffin, USA; 4 Department of Endocrinology, Diabetes, and Metabolism, HealthPartners, Minneapolis, USA

**Keywords:** anaplastic thyroid cancer, braf v600e mutation, chemotherapy adverse effects, guillain barre’s syndrome (gbs), thyroid neoplasm

## Abstract

BRAF V600E mutation in thyroid malignancies is associated with an aggressive phenotype with more rapid tumor growth and higher mortality. V600E is a driver mutation in the BRAF proto-oncogene, where valine (V) is substituted by glutamic acid (E) at amino acid 600. New chemotherapeutic agents targeting the mitogen-activated protein kinase (MAPK) pathway, including direct BRAF inhibitors, have become available and are increasingly used in various advanced thyroid malignancies. These agents are associated with various rare neurological adverse effects. We present a case of Guillain-Barre syndrome (GBS) secondary to dabrafenib and trametinib therapy for the management of anaplastic thyroid carcinoma. A few cases of GBS have been reported previously with the use of these agents in the treatment of melanoma. To our knowledge, this is the first case of GBS with the use of dabrafenib and trametinib for advanced thyroid malignancy. The knowledge of this rare, potentially life-threatening condition is important for clinicians to know, given the increased use of these agents in managing advanced thyroid malignancies.

## Introduction

Advanced dedifferentiated or poorly differentiated thyroid carcinomas (DTCs) refractory to radioactive iodine therapy and anaplastic thyroid carcinoma (ATC) are associated with a significantly higher mortality rate compared to locoregional DTC [1]. Traditionally, there have been limited options for treating these cases. These options have included multikinase inhibitors (lenvatinib and sorafenib), which are poorly tolerated due to the development of considerable side effects leading to discontinuation in most cases [2]. Advancements in our understanding of underlying mutational pathways in such carcinomas have led to the discovery and use of targeted therapies, which have shown promising results with better tolerability profiles. Dabrafenib (BRAF inhibitor) and trametinib (MEK inhibitor) are now approved by the Food and Drug Administration( FDA) for the management of ATC harboring BRAF V600E mutation [3]. These agents have also shown promising results in managing metastatic DTC with BRAF V600E mutations [4]. The BRAF V600E mutation is present in almost 45% of papillary thyroid carcinomas, 15% of poorly DTCs, and 20% of ATCs [3]. BRAF V600E mutation in thyroid malignancies is associated with an aggressive phenotype with more rapid tumor growth and higher mortality [5]. With the increased use of these agents for managing advanced thyroid malignancies, clinicians should be aware of rare but potentially life-threatening side effects associated with these medications. To our knowledge, this is the first case of Guillain-Barre syndrome (GBS) in a patient treated with the combination of dabrafenib and trametinib for advanced thyroid malignancy.

## Case presentation

**Figure 1:**
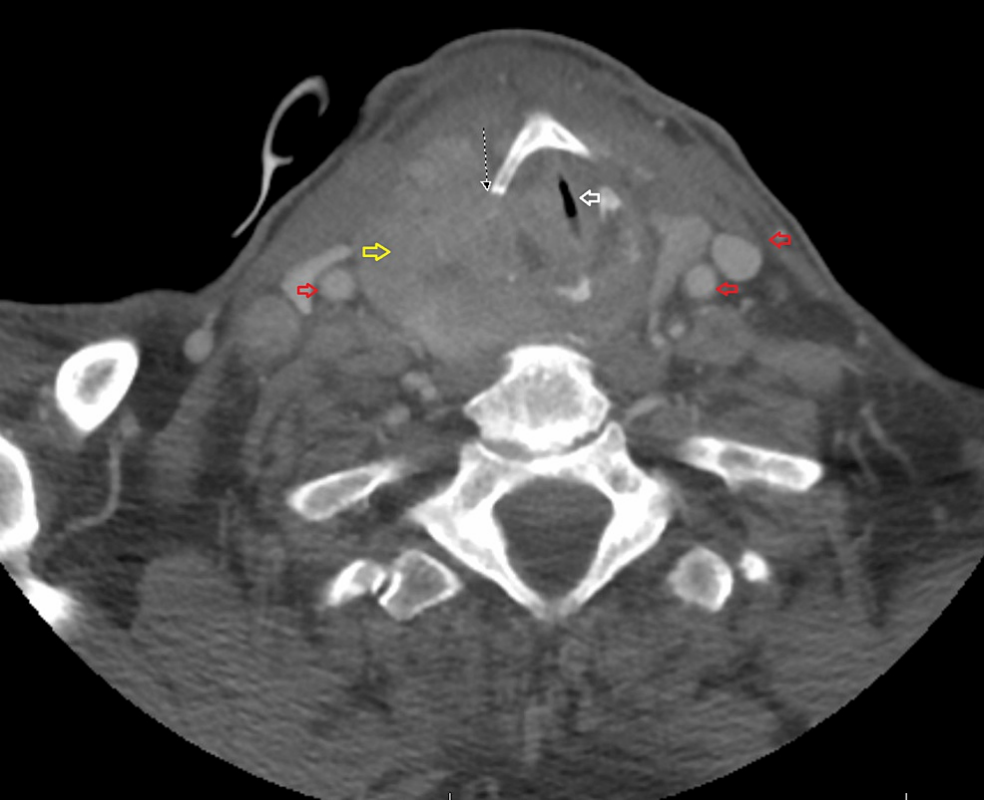
Cross-sectional view showing the large right mass causing left-sided tracheal deviation (yellow area, thyroid mass; red arrow, enlarged cervical lymph nodes; and white arrow, tracheal deviation).

We present a case of a 75-year-old male who initially presented for stridor evaluation. His medical history was significant for hypertension, type 2 diabetes, and gastroesophageal reflux disease. His medications included metformin, pantoprazole, and acetaminophen. He underwent computed tomography (CT) scan of the neck, which showed a right-sided thyroid mass with the invasion of the thyroid cartilage, cricoid cartilage, and multiple anterior tracheal rings (Figure 1). He underwent a biopsy of the mass, and pathology revealed a small focus of tall-cell variant of papillary thyroid carcinoma with squamous cell features concerning ATC. Molecular analysis was positive for BRAF and TERT mutations. Surgical resection of the mass was attempted, but the tumor was deemed inoperable due to extensive tracheal invasion. Tracheal biopsies were obtained, which revealed invasive carcinoma with squamous differentiation, representing anaplastic thyroid carcinoma. Further molecular analysis revealed that in addition to the BRAF and TERT mutations seen in the previous biopsies, the carcinoma in the right trachea harbors a PIK3CA mutation, while the carcinoma of the right neck had a PTEN mutation. This is suggestive of molecular progression. Positron emission tomography (PET)/CT scan revealed FDG avid thyroid mass with increased uptake in the thyroid cartilage, cricoid cartilage, cervical trachea, and multiple bilateral cervical lymph nodes. No distant metastasis was noted on PET/CT.

Due to the inoperability of the tumor (Stage IVB), which harbored a BRAF V600E mutation, the patient was started on chemotherapy with dabrafenib 150 mg twice daily and trametinib 2 mg daily. Three weeks after the initiation of therapy, he presented to the hospital with fatigue and weakness in his lower extremities for three days which progressed to a feeling of heaviness in his upper extremities. He was unable to ambulate without support at the time of the presentation. He denied any bladder and bowel dysfunction. He did not complain of any difficulty in breathing. There was no history of significant falls, trauma, or recent illnesses. Vitals signs were normal. On examination, the patient was conscious and fully oriented. Evaluation of cranial nerves I-XII was normal. He had a strength of 2/5 in bilateral lower extremities and 4/5 in bilateral upper extremities. Reflexes were absent in the lower extremities. The sensation was intact to light touch and pinprick bilaterally. The vibration was absent in the feet and knees bilaterally and normal in the hands. Blood count was normal, erythrocyte sedimentation rate (ESR) was 18 (0-22 mm/hour), and C-reactive protein (CRP) was 0.9 (0.3-1.0 mg/dL). Neurology was consulted. Magnetic resonance imaging (MRI) of the brain did not reveal any acute intracranial process. Mild chronic microvascular ischemic changes were noted with mild generalized cerebral atrophy. MRI of the spine revealed a normal appearance of the spinal cord, no significant central stenosis, or neuroforaminal narrowing. There was no evidence of metastatic disease. CT chest revealed developing pneumonia in the left lower lobe, likely secondary to aspiration. This CT scan did not show any change in the size of the thyroid mass or any further invasion of neighboring structures. A lumbar puncture was performed. Cerebrospinal fluid (CSF) analysis showed a cell count of 33 cells/µL (0-5 cells/µL), protein of 43 mg/dL (20-40 mg/dL), and glucose of 65 mg/dL (50-80 mg/dL). Thiamine levels were low <6 nmol/L (8-30 nmol/L). Other tests conducted were as follows: copper level was 112 µg/dL (62-140 µg/dL), vitamin B6 was 21 µg/L (5-50 µg/L), and vitamin B12 was 440 pg/dL (150-960 pg/dL). Lyme antibody immunoassay was negative (<0.9 AI). Electromyography could not be obtained.

With rapid onset quadriparesis, proximal more than distal, and areflexia, his presentation was most concerning for GBS. As there have been previous case reports of GBS with BRAF inhibitor therapy, dabrafenib and trametinib were held. No further microbiological testing was performed in the absence of any history or signs of any recent or current infections. The patient was started on intravenous immunoglobulin (IVIG) 55 gm daily for five days (2 mg/kg). The patient’s weakness started improving gradually, and he was subsequently transferred to the inpatient rehabilitation unit.

One month after his initial presentation with GBS, a decision was made to rechallenge him with dabrafenib and trametinib therapy. After two months, he was found to have progression of his locoregional disease and the development of new pulmonary and mediastinal metastases. He developed dysphagia and needed the placement of a feeding tube and photodynamic therapy. His clinical condition continued to deteriorate, and a decision was made to place him on hospice care as he was not a candidate for other systemic therapies. He passed away after a few weeks.

## Discussion

GBS is an infrequent but potentially life-threatening complication associated with dabrafenib and trametinib therapy. Early diagnosis is essential for early treatment institutions to avoid progression and potential involvement of the respiratory muscles. This complication has been previously reported with the use of these drugs in the treatment of advanced BRAF V600E-positive melanoma [6,7]. Other BRAF and MEK inhibitor combinations include encorafenib-binimetinib and vemurafenib-cobimetinib [8]. A phase 2 trial for metastatic radioiodine refractory thyroid carcinoma treatment using vemurafenib and cobimetinib has already been completed, and a phase 2 trial using encorafenib and binimetinib therapy is ongoing [8]. A pharmacovigilance study reported an incidence of GBS to be 0.05% with the use of dabrafenib and trametinib in the treatment of melanoma [9]. In this study, the incidence of GBS was higher with other agents. About 0.1% had GBS with vemurafenib and cobimetinib, and 0.5% with vemurafenib and cobimetinib [9]. Based on our review of existing literature, this is the first case of GBS with the use of dabrafenib and trametinib in treating advanced thyroid malignancy.

Our patient’s development of GBS was categorized as grade 3 using the Common Terminology Criteria for Adverse Events v5.0 (CTCAE) [10]. Patients meeting the clinical diagnostic criteria for GBS should undergo a lumbar puncture (LP) to support the diagnosis. LP will typically show an albuminocytologic dissociation with elevated protein and normal cell count on CSF analysis. Normal CSF protein and mild pleocytosis (<50 cells/µL) are not uncommon in patients with GBS [11]. Nerve conduction studies and electromyography can be used to support the diagnosis. Other advanced tests can be done in patients with atypical clinical features. Patients with GBS should be admitted to the inpatient setting for monitoring and therapy. Mainstay therapies include IVIG or plasma exchange therapy. Systemic steroid therapy has not been shown to improve outcomes and should be avoided. The long-term prognosis for recovery and independent ambulation remains favorable for most patients with GBS [12].

Whether these patients who develop GBS with dabrafenib and trametinib can be rechallenged with these agents remains unclear. In a previously reported case, the patient redeveloped GBS after reexposure to dabrafenib to treat melanoma [6]. In another reported case of development of acute motor and sensory axonal neuropathy (AMSAN) variant of GBS with dabrafenib and trametinib, the patient was rechallenged with another BRAF + MEK inhibitor combination (vemurafenib and cobimetinib), leading to the redevelopment of GBS [7]. Our patient was rechallenged with dabrafenib and trametinib therapy and did not develop GBS again but had a progression of primary carcinoma. There is no consensus on the timeline for developing GBS with these agents.

GBS is an acute immune-mediated polyneuropathy. The pathogenesis of GBS with the use of targeted kinase inhibitors is not well understood. The MAPK signaling pathways perform multiple complex cellular functions. They elicit different physiologic responses to various external factors leading to cell proliferation, differentiation, development, inflammatory responses, and apoptosis [13]. The study of the transcriptional profile of the leukocytes in patients with GBS has shown upregulation of the MAPK signal transduction pathway [14]. These pathways also play a critical role in the nervous system, including axonal maintenance, transport, and regulation of cells involved in neuronal myelination [15].

Activation of the mitogen-activated protein kinase (MAPK) pathway is associated with various thyroid malignancies [16]. Genetic alterations in this pathway have become attractive targets for therapy. Dabrafenib targets the BRAF in this pathway, and trametinib targets the MEK downstream of the BRAF in the pathway [17]. This drug combination results in dual inhibition of the MAPK pathway.

The combination therapy of dabrafenib and trametinib has been shown to improve the duration of response and overall survival in 36 patients enrolled in the Rare Oncology Agnostic Trial (ROAR) basket trial [18]. In a small clinical trial of 10 patients with advanced dedifferentiated thyroid carcinoma, short-term use of dabrafenib was shown to promote redifferentiation of the tumor with the renewal of the ability to concentrate radioactive iodine. This led to partial response or stability of disease six months after radioactive iodine treatment in 6 out of 10 patients [19]. The use of dabrafenib and trametinib in patients with progressive radioactive iodine refractory papillary thyroid carcinoma with BRAF V600E mutation led to objective responses in 54% of the patients with a median follow-up of 13 months [20]. 

## Conclusions

With the increased clinical use of BRAF and MEK inhibitors in the management of advanced thyroid malignancies, clinicians need to be cognizant of the potentially life-threatening side effects associated with these therapeutic agents.
